# Staghorn renal stones: what the urologist needs to know

**DOI:** 10.1590/S1677-5538.IBJU.2020.99.07

**Published:** 2020-09-02

**Authors:** Fabio C. M. Torricelli, Manoj Monga

**Affiliations:** 1 Hospital das Clínicas Faculdade de Medicina Universidade de São Paulo SP Brasil Divisão de Urologia, Hospital das Clínicas, Faculdade de Medicina da Universidade de São Paulo, SP, Brasil;; 2 Streem Center for Endourology & Stone Disease Glickman Urological & Kidney Institute The Cleveland Clinic ClevelandOH USA Stevan B, Streem Center for Endourology & Stone Disease, Glickman Urological & Kidney Institute, The Cleveland Clinic, Cleveland, OH, USA

**Keywords:** Infections, Kidney, Urinary Calculi

## Abstract

Patients with staghorn renal stones are challenging cases, requiring careful preoperative evaluation and close follow-up to avoid stone recurrence. In this article we aim to discuss the main topics related to staghorn renal stones with focus on surgical approach. Most of staghorn renal stones are composed of struvite (magnesium ammonium phosphate) and are linked to urinary tract infection by urease-producing pathogens. Preoperative computed tomography scan and careful evaluation of all urine cultures made prior surgery are essential for a well-planning surgical approach and a right antibiotics choice. Gold standard surgical technique is the percutaneous nephrolithotomy (PCNL). In cases of impossible percutaneous renal access, anatrophic nephrolithotomy is an alternative. Shockwave lithotripsy and flexible ureteroscopy are useful tools to treat residual fragments that can be left after treatment of complete staghorn renal stone. PCNL can be performed in supine or prone position according to surgeon’s experience. Tranexamic acid can be used to avoid bleeding. To check postoperative stone-free status, computed tomography is the most accurate imaging exam, but ultrasound combined to KUB is an option. Intra-operative high-resolution fluoroscopy and flexible nephroscopy have been described as an alternative for looking at residual fragments and save radiation exposure. The main goals of treatment are stone-free status, infection eradication, and recurrence prevention. Long-term or short-term antibiotic therapy is recommended and regular control imaging exams and urine culture should be done.

## INTRODUCTION

Staghorn renal stones are large kidney stones that fill the renal pelvis and at least one renal calyces. Most of times they are composed of struvite (magnesium ammonium phosphate), which are linked to recurrent urinary tract infections by urease-producing pathogens. In developing countries, 10 to 15% of all urinary calculi are struvite stones and women are twice more frequently affected than men. In developed countries its incidence is lower due to early diagnosis and management of renal stones ( 1 - 3 ). Majority of cases are unilateral, but up to 15% of cases may have both kidneys affected ( 4 ). Factors that predispose patients to struvite stones include female gender, extremes of ages, congenital urinary tract malformations, urinary stasis, urinary diversion, neurogenic bladder, indwelling Foley catheters, distal renal tubular acidosis, medullary sponge kidney, and diabetes mellitus ( 1 - 5 ). Significant morbidity and potential mortality of staghorn stones make prompt assessment and treatment mandatory.

In this article, we aim to discuss major points related to staghorn renal stone, including its pathogenesis, management, and prevention. Main focus will be on surgical approach and its results.

### Pathogenesis

Struvite stone formation is associated with bacteria that produce the enzyme urease, including both gram-positive and gram-negative species, such as Proteus, Staphylococcus, Pseudomonas, Providencia, and Klebsiella. But not every strain produces the urea-splitting enzyme. While 100% of Proteus spp, Providencia spp, and Morgenalla morganii spp produce the urea-splitting enzyme, not all Klebsiella spp and Staphylococcus spp are able to produce urease. Despite being a major cause of urinary tract infection, only 1.4% of Escherichia coli spp are able to produce urease and are, therefore, not considered a major cause of struvite stone formation ( 1 , 6 ). Parkhomenko et al. evaluated stone culture and urine culture in 1191 patients who underwent PCNL and found that while stone cultures were positive in 72% of patients with struvite stones, urea-splitting organisms accounted for only half of these positive exams. Remarkably, most of pathogens were resistant to first- and second-generation cephalosporins. And looking at to prior urine exams, two-thirds of struvite formers with negative stone culture had at least one positive culture for a urea-splitting organism on urine culture going back 1 year from the time of surgery ( 7 ). These findings have important implications and should be taken into account when choosing preoperative antibiotics before PCNL. We recommend a careful evaluation of all urine culture before surgery.

In order to identify the casual pathogen, stone culture is the best way to identify urease-producing bacteria. In the absence of stone culture, urine should be sampled from the kidney at the time of surgery.

Struvite kidney stones formation is associated with an increase in urinary pH in the presence of urease-producing bacteria. Pathogens who produce urease enzyme split urinary urea into ammonia, which is hydrolyzed to bicarbonate and ammonium. Then, these will form magnesium ammonium phosphate and carbonate apatite upon binding to cations. Bacteria also metabolize the citrate in urine and stop its protective binding to calcium and phosphate ( 5 , 6 , 8 ).

Currently, it is increasing the number of staghorn calculi that grow up in the absence of infection. Winoker et al. evaluated 25 patients with staghorn renal stones with no infection and compared to 64 usual staghorn stones (infection stones) in terms of medical comorbidity, 24-hour urine parameters, stone and urine microbiology, stone compositions, and intraoperative findings. Hyperoxaluria was significantly higher in patients with no infection and was the only significant finding of the study. Authors concluded that it is not clear why some metabolic stones assume staghorn configuration, but probably it is not influenced by standard determinants of stone development ( 9 ).

### Management

The gold-standard surgical treatment for staghorn renal stones is the same for most of kidney stones size bigger than 2.0cm, which is the percutaneous nephrolithotomy (PCNL). Although a complete stone-free postoperative status with only one session is a hard achievement when treating a complete staghorn stone, a well-planned approach (staged or not) may lead to very satisfactory outcomes. In a systematic review and meta-analysis comparing PCNL with retrograde intrarenal surgery, authors concluded that PCNL was associated with higher stone-free rate, but also with a higher complication rate and blood loss ( 10 ).

In one of the first case-series reporting PCNL outcomes for staghorn stones, authors reported complete stone clearance rates of 98.5% and 71% for partial and complete staghorn stones, respectively. The absence of computed tomography as imaging control exam is one major limitation and may have super estimated the stone-free rate. The overall complication rate in this study was as low as 4% ( 11 ). In a prospective, randomized, single center study involving 50 kidneys with complete staghorn calculi, 27 renal units were treated with SWL monotherapy and 23 were treated with combined PCNL and SWL. Combined approach led to a higher stone-free rate and a lower complication rate ( 12 ). Another prospective, randomized trial comparing PCNL versus open surgery for complete staghorn stones also found favorable outcomes for PCNL, mainly in terms of complication rate. Forty-three patients who underwent PCNL were compared to 45 patients who underwent open surgery. Intraoperative complications including bleeding requiring blood transfusion (16.3% vs. 37.8%) and major postoperative complications including massive hematuria requiring blood transfusion, sepsis, urinary leakage and wound infection (18.6% vs. 31.1%) were significantly higher with open approach. PCNL was also associated with a significant shorter operative time (127 vs. 204 min), shorter hospital stay (6.4 vs. 10 days) and earlier return to work (2.5 vs. 4.1 weeks) ( 13 ).

In a comparison between PCNL and laparoscopic and open surgery (anatrophic nephrolithotomy), authors reported that PCNL was associated with the lowest stone-free rate (43.75%) compared to the laparoscopic (80%) and open surgery (92.85%). However, after a mean follow-up period of 12.1 months, technetium-99 dimercaptosuccinic acid renal scintigraphy revealed that the decrease in the renal function was greater in the open approach (-8.66) compared to the laparoscopic (-6.04) and PCNL (-2.12) techniques ( 14 ). In a recent systematic review and meta-analysis comparing PCNL and open surgery authors found that although initial stone-free rate of open surgery was better, final stone-free rate after ancillary procedures was similar. As advantage, PCNL was associated with lower overall complication rate, shorter operative time, shorter hospitalization time, less blood loss and blood transfusion compared with open surgery ( 15 ).

Regarding PCNL technique, there are studies comparing the outcomes from prone and supine PCNL for staghorn stones. A Clinical Research Office of the Endourological Society (CROES) study including 1079 prone PCNL and 232 supine PCNL for staghorn stones management showed a shorter operative time and a higher stone-free rate with prone position, whereas complication rate was similar. The main criticism of this study is the heterogeneity of data from different centers ( 16 ). In a recent study data of patients who underwent PCNL for staghorn stones in supine or prone position by a single urologist were prospectively collected. Seventy-eight cases were enrolled, 39 supine PCNL and 48 prone PCNL similar for demographic and stone-related characteristics were compared. Stone-free rate was similar between the groups (64.1% supine vs. 60.4% prone), however, supine PCNL was associated with shorter operative time and a lower hemoglobin drop ( 17 ). We believe that patient’s position is not the main key point related to stone clearance and surgical complications, thus urologists should perform the technique that they are more familiarized.

As PCNL in the treatment of staghorn stones has been linked to significant bleeding, some authors have proposed the use of tranexamic acid to prevent or minimize this complication. Mohammadi et al. in a randomized controlled trial including 120 patients with staghorn calculi divided cases to receive either 1g of tranexamic acid intravenously or normal saline. The mean volume of blood loss was significantly higher in the control group patients than in those receiving tranexamic acid, however there was no difference in the transfusion rate between the groups ( 18 ). Although no significant decrease in transfusion rate was found, previous studies have already demonstrated the advantages of administration of tranexamic acid before PCNL ( 19 , 20 ). Kumar S et al. in a prospective study including 200 patients who underwent PCNL randomized the cases in 2 equal groups. Patients in the tranexamic acid group received 1g of tranexamic acid at induction followed by 3 oral doses of 500mg during 24 hours, while those in the control group did not receive the drug. Mean hemoglobin decrease (1.39 vs. 2.31mg/dL) and blood transfusion rate (2% vs. 11%) were significant lower in the tranexamic acid group. The stone clearance rate was similar in both groups (91% vs. 82%), while complication rate (33% vs. 59%) was again significantly lower in the interventional group ( 20 ). Based on these findings, we recommend the use of tranexamic acid in PCNL for starghorn stones in patients with no contraindications for the medication (i.e. renal insufficiency).

As PCNL in complete staghorn stones is a challenging procedure, some authors tried to use nomograms to assist urologists to predict surgical outcomes. Sfoungaristos et al. compared the accuracy of Guy’s, CROES and STONE nomograms for staghorn stones and found that STONE was the only significant independent predictor in multivariate analysis. STONE also revealed the highest predictive accuracy compared to Guy’s and CROES nomogram ( 21 ). Choi et al. in a study with 305 PCNL for staghorn stones also compared the predictive value and accuracy of those three stone-scoring systems for the treatment success of staghorn stone. Again, only STONE monogram was significantly associated with surgical outcomes. On a multivariate logistic regression analysis, independent predictors for stone-free rate were number of involved calices, STONE nephrolithometry, and pre-existent urinary tract infection ( 22 ).

After PCNL, imaging control exams should be performed to check stone-free status or to identify residual fragments. Usually, computed tomography scan or association of ultrasound and KUB is performed. In order to save radiation and costs of CT scan, which is the most accurate imaging postoperative exam, Portis et al. evaluated the efficiency intraoperative high-resolution fluoroscopy and flexible nephroscopy in combination after PCNL in 25 kidneys. Of 21 renal units considered endoscopically and fluoroscopically stone-free, postoperative CT demonstrated that only 6 had residual fragments, of which all were <4mm. Intraoperative fluoroscopy after nephroscopy demonstrated fragments in 36% of renal units, of which after further nephroscopy 78% were stone-free on CT scan. Authors concluded that high-resolution fluoroscopy and flexible nephroscopy together present high accuracy to find residual fragments, allowing its treatment concomitantly or in a second procedure ( 23 ).

Others surgical procedures to stone removal have few and/or specific indications for staghorn renal stones management. Shock wave lithotripsy (SWL) should be considered only for treatment of residual fragments, as SWL has been associated with several potential complications when used to treat large staghorn calculi, including sepsis, obstructive nephropathy from steinstrasse, renal colic, and perinephric hematoma. Ureteroscopy should be used for residual fragments treatment or combined to PCNL to decrease number of punctures. And, currently, open surgery is reserved to rare circumstances when PCNL is not available or cannot be safely performed due to anatomical abnormalities such as a pelvic kidney, retro-renal colon, or spinal deformities that make hard percutaneous access to the kidney ( 1 ). The AUA guidelines recommend anatrophic nephrolithotomy in patients for whom treatment of a struvite staghorn calculus is not likely to be successful with a “reasonable” number of PCNL or SWL ( 24 , 25 ).

New technologies are getting enrolled to the endourologist armamentarium to decrease postoperative complications in PCNL for staghorn stones. In an initial trial with 12 patients, a three-dimensional (3D) printing model for preoperative planning in the treatment of complete staghorn stones was used. The rationale was that with repeated simulations before surgery, surgeon could be more familiar with the anatomy and angle between the renal calyxes, and ultimately, it could improve surgical outcomes. In this study, a 3D printing technology to create a patient-specific model based on preoperative computed tomography scan in prone position was performed. Then, a model for preoperative planning and in vitro full immersion simulation was done. Next, the puncture that yielded the best stone-free rate was selected on the model and translated to the actual patient. Authors have found that there was a high degree of correlation between the best simulation and the actual postoperative results ( 26 ).

### Preventing recurrence

Once infection stones are identified, three principles exist for their treatment. Firstly, all stone burden should be removed. Secondly, antibiotics should be used to treat the infection, aiming sterilize the urine. Thirdly, recurrence should be prevented ( 6 ).

Antibiotics are clearly important for the safe management of infection stones, but guidelines for the timing and duration of therapy have not yet been established. EAU has issued a grade B recommendation that long-term or short-term antibiotic therapy should be given to all patients with infection stones ( 27 ). Similarly, the AUA has recommended that all staghorn calculi should be assumed to be struvite and should be treated with prophylactic or suppressive antibiotic therapy ( 24 , 25 ). However, currently, there is no high-level evidence for specific antibiotic regimens. Iqbal et al. in a retrospective study including 43 patients with struvite stone who underwent PCNL reported an initial stone-free rate of 42%. Stone recurrence was noted in 23% of patients and it was more important in patients with residual fragments. Interestingly, 60% of patients with residual fragments had their stone stable with no growth after a median follow-up of 22 months under antibiotic prophylaxis. In this study, independent predictors of stone activity included the presence of residual stones >0.4cm2, preoperative large stone burden (>10cm2), and the presence of medical comorbidities ( 28 ). As recommendation, we suggest the use of antibiotics in the presence of stone fragments, which might later require treatment with several different modalities (ureteroscopy, SWL, or repeat PCNL) to achieve complete stone clearance. Repeat imaging and urine cultures should be performed periodically each 3 to 6 months to check stone-free status or identify recurrence ( 1 ).

**Figure 1:**
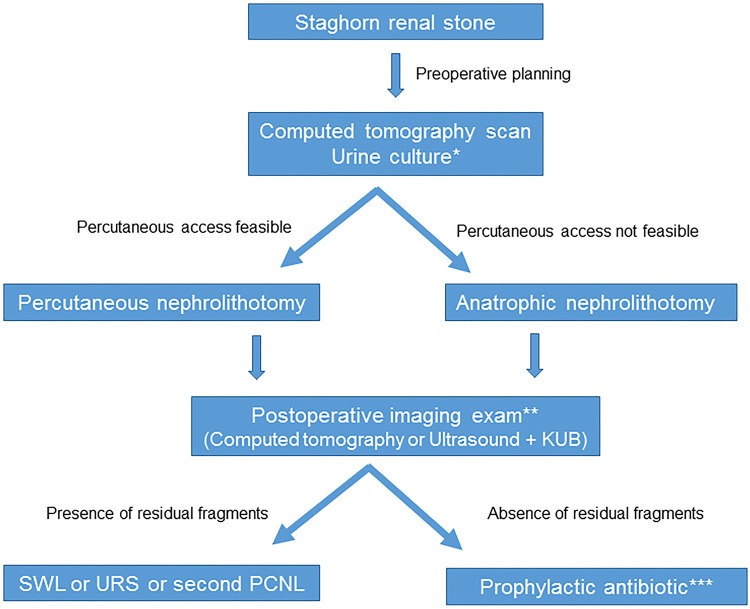
Summarizes staghorn renal stone treatment.

Urease inhibitors have demonstrated only modest benefit for the treatment of struvite stones. Griffith et al. used acetohydroxamic acid in a randomized double-blind study of 94 patients with struvite stones and chronic urinary tract infection. Stone growth occurred in 17% of the acetohydroxamic acid group and in 46% of the placebo group. Although the recurrence rate was significantly lower, side effects were judged ‘intolerable’ in 22.2% of patients in the acetohydroxamic acid group compared to only 4.1% in the placebo group ( 29 ). Others studies had similar findings in preventing stone recurrence, but adverse effects such as tremulousness and phlebothrombosis have limited its use ( 30 , 31 ).

Urinary acidification with agents such as ascorbic acid, ammonium chloride, ammonium sulphate, ammonium nitrate, and methionine has been used to clear residual fragments and to prevent future stone formation following stone clearance. However, it can be difficult to maintain acidification of the urine with these agents, particularly in the presence of infection ( 1 ).

## CONCLUSIONS

Staghorn renal stones are most of times composed of struvite and related to urinary tract infection. Careful preoperative planning is essential to achieve stone-free status. PCNL is the treatment of choice and auxiliary procedures such as SWL and flexible ureteroscopy should be used to treat residual fragments. Both prone and supine are effective. Tranexamic acid before PCNL seems to decrease surgical bleeding. The goals of the treatment are the complete absence of kidney stones and eradication of infection with antibiotics. Close follow-up is advised with regular imaging exams and urine culture.
